# Predicting maximal oxygen uptake from a 3-minute progressive knee-ups and step test

**DOI:** 10.7717/peerj.10831

**Published:** 2021-03-15

**Authors:** Yu-Chun Chung, Ching-Yu Huang, Huey-June Wu, Nai-Wen Kan, Chin-Shan Ho, Chi-Chang Huang, Hung-Ting Chen

**Affiliations:** 1 Center of General Education, Taipei Medical University, Taipei, Taiwan; 2 Service Systems Technology Center, Industrial Technology Research Institute, Hsinchu, Taiwan; 3 Department of Combat Sports and Chinese Martial Arts, Chinese Culture University, Taipei, Taiwan; 4 Graduate Institute of Sports Science, National Taiwan Sport University, Taoyuan, Taiwan; 5 Physical Education Office, Ming Chuan University, Taipei, Taiwan

**Keywords:** Aerobic ability, 3-min Harvard step test, Cardiovascular function, Field tests

## Abstract

**Background:**

Cardiorespiratory fitness assessment is crucial for diagnosing health risks and assessing interventions. Direct measurement of maximum oxygen uptake (V̇O_2_ max) yields more objective and accurate results, but it is practical only in a laboratory setting. We therefore investigated whether a 3-min progressive knee-up and step (3MPKS) test can be used to estimate peak oxygen uptake in these settings.

**Method:**

The data of 166 healthy adult participants were analyzed. We conducted a V̇O_2_ max test and a subsequent 3MPKS exercise test, in a balanced order, a week later. In a multivariate regression model, sex; age; relative V̇O_2_ max; body mass index (BMI); body fat percentage (BF); resting heart rate (HR0); and heart rates at the beginning as well as at the first, second, third, and fourth minutes (denoted by HR0, HR1, HR2, HR3, and HR4, respectively) during a step test were used as predictors. Moreover, *R^2^* and standard error of estimate (SEE) were used to evaluate the accuracy of various body composition models in predicting V̇O_2_max.

**Results:**

The predicted and actual V̇O_2_ max values were significantly correlated (BF% model: *R^2^* = 0.624, SEE = 4.982; BMI model: *R^2^* = 0.567, SEE = 5.153). The BF% model yielded more accurate predictions, and the model predictors were sex, age, BF%, HR0, ΔHR3−HR0, and ΔHR3−HR4.

**Conclusion:**

In our study, involving Taiwanese adults, we constructed and verified a model to predict V̇O_2_ max, which indicates cardiorespiratory fitness. This model had the predictors sex, age, body composition, and heart rate changes during a step test. Our 3MPKS test has the potential to be widely used in epidemiological research to measure V̇O_2_ max and other health-related parameters.

## Introduction

In 2016, the American Heart Association launched a series of publications promoting the clinical evaluation of cardiorespiratory fitness (CRF) with the overall aim of improving the prevention and treatment of cardiovascular disease (CVD; Ross et al., 2016). Furthermore, the association urged the US federal government to compile a registered CRF database (Kaminsky et al., 2013); this highlights the importance of CRF. CRF is generally defined as the integrated ability to transport oxygen from the atmosphere to the mitochondria for physical activity. Notably, CRF involves the respiratory, circulatory, and neuromuscular systems and has a clear and direct relationship with the functions of various systems. Individuals with weak CRF have an up to 70% all-cause mortality rate and 56% cardiovascular mortality rate (Kodama et al., 2009). Similarly, every 1-MET increase in athletic ability reduces all-cause mortality and cardiovascular mortality rates by 15% and 13%, respectively (Kodama et al., 2009). Numerous studies have suggested that CRF and CVD are related to all-cause mortality and cancer mortality (Blair et al., 1989; Laukkanen et al., 2004; Sui, LaMonte & Blair, 2007; Sawada et al., 2014; Sui, LaMonte & Blair, 2007). A recent meta-analysis reported CRF to be a predictor of the risk of sudden cardiac death (Jiménez-Pavón, Lavie & Blair, 2019). Therefore, CRF assessment is crucial for diagnosing health risks and assessing interventions.

CRF can be measured using the respiratory data of exercising participants. Specifically, these data are used to calculate maximal oxygen uptake (V̇O_2_ max), the gold standard for CRF measurement; in the measurement, participants either run on a treadmill or use an ergometer at an exercise intensity that increases progressively until a given maximum is reached. Although submaximal exercise models and nonexercise models (without an exercise test) are alternatives for estimating V̇O_2_ max in measuring CRF (Abut, Akay & George, 2016), the direct measurement of V̇O_2_ max yields more objective and accurate results. However, such measurement is inconvenient because it requires expensive equipment and well-trained experimenters. In addition, participants perceive such measurement tests to be exhausting, time-consuming, and relatively risky and are thus less willing to participate. Accordingly, researchers have developed various submaximal exercise tests to indirectly estimate V̇O_2_ max; moreover, retrospective studies conducted by the American Heart Association have demonstrated that CRF indicators, whether directly measured or indirectly estimated, are robust indicators of health (Ross et al., 2016).

Submaximal exercise is a common method for estimating V̇O_2_ max, particularly in epidemiological research and large-scale physical fitness testing that involve numerous participants. The field tests in these measurement procedures include running, shuttle running, and the step test, with the step test being the most common method for evaluating cardiovascular function (Grant, Joseph & Campagna, 1999). In particular, the YMCA step test is widely used to predict V̇O_2_ max (Beutner et al., 2015). Currently, the Sports Administration of Taiwan’s Ministry of Education uses the 3-min Harvard step test for its National Physical Fitness and Cardiovascular Test. Specifically, three heart rate measurements are used to calculate the step-up index. However, previous studies have reported considerable differences in the validity of using the step test index to evaluate V̇O_2_ max, with the corresponding correlation coefficient (R) being 0.35–0.94 (Buckley et al., 2004; Chang & Lin, 1995; Mazic et al., 2001; Su, Lin & Hsieh, 2006; Chang & Lin, 1995; Yoopat, Vanwonterghem & Louhevaara, 2002). Furthermore, step tests require the use of step-up boxes, and the overall test time must be at least 6 min to allow for heart rate recovery. Participants who are less physically fit or who have knee conditions may find it difficult to complete the test and may also fall in the process of going up and down the stairs. A team of Japanese researchers developed a new 3-min walking test (Cao et al., 2013). Specifically, their main evaluation criteria comprised participant characteristics such as age, sex, and BMI as well as participants’ RPE during exercise. These criteria were determined to be effective predictors of V̇O_2_ max, and participants thought that this method was quicker and easier.

Tests of general CRF are crucial to the clinical evaluation of CVD. Additionally, the advantages and disadvantages, such as venue size, participant willingness, and the instruments, of various past field tests should be considered during the formulation of new methods, as done in the present study. Accordingly, we conducted the present study with the aim of developing a rapid, convenient, and low-risk model that can predict V̇O_2_ max in Taiwanese adults. Additionally, our model accords with the principle that physical exercise ought to be progressive. We investigated the feasibility of using a 3-min progressive knee-ups and step (3MPKS) test to predict V̇O_2_ max.

## Materials and Methods

### Participants

Prospective participants were excluded if they (1) had cardiovascular, pulmonary, or metabolic diseases; (2) had neurological, muscular, or skeletal disorders that affected their athletic ability; (3) had other health conditions that made them unsuited for moderate or intense exercise; or (4) were taking medications that could affect the outcome of this study. In total, among 200 participants recruited for this experiment, 166 completed the test. The data of the 166 participants were included in the analysis (age: 20–64 years; 65 men, 101 women). Among the 34 participants excluded, one participant withdrew from the experiment after experiencing suspected symptoms of arrhythmia during exercise; 11 were excluded because they failed to complete the step test within the requisite time (3 min); 12 were excluded because they could not attain the requisite step frequency and knee height for 20 consecutive seconds; nine were excluded because they had missing or improperly measured heart rate data; and one was excluded for having a “0” in their heart rate data. All participants signed an informed consent form after understanding their rights, the risks when participating in this study, and the purpose and method of our research. Our research plan was approved by the Institutional Review Boards (IRBs) of the Industrial Technology Research Institute and of Taipei Medical University (IRB No: N201808055). Participant characteristics are detailed in Table 1.

**Table 1:** Participant characteristics.

	Total	Training dataset	Testing dataset
Sample size(n)	166	124	42
Age(years)	41.9 ± 9.6	42.2 ± 9.4	40.8 ± 10.2
Male (n)	65	44	21
Height	164.83 ± 8.35	164.33 ± 8.07	166.30 ± 9.06
Weight	65.63 ± 13.60	65.22 ± 14.08	66.85 ± 12.15
Body fat (%)	27.81 ± 7.92	27.79 ± 7.65	27.86 ± 8.75
V̇O_2_ max (ml kg^−1^ min^−1^)	34.45 ± 8.69	34.06 ± 8.14	35.61 ± 10.15
HR0	86.04 ± 12.78	86.04 ± 12.99	86.02 ± 12.29
ΔHR3- HR0	71.00 ± 13.24	71.10 ± 13.41	70.69 ± 12.87
ΔHR3-HR4	14.64 ± 13.72	14.14 ± 13.94	16.65 ± 14.09

**Notes.**

Data are presented as mean ± standard deviation.

HR0 heart rate at the beginning ΔHR3-HR0 difference between third minute heart rate and beginning heart rate ΔHR3-HR4 difference between third minute and fourth minute heart rates

### Procedure

The anthropometric and body composition measures were height, weight, and body fat percentage (BF%). BF% was measured using bioelectrical impedance analysis (InBody 720, Biospace, USA; McLester et al., 2020), and body mass index (BMI, in kg/m^2^) was calculated as the quotient that is weight (in kilograms) divided by the squared height (in meters).

We conducted two exercise tests in a counterbalanced design. The second test was conducted exactly 1 week after the first and at the same time of the day to ensure that the participants recovered adequately from the first exercise. The participants underwent 5–10 min of dynamic warm-up prior to both exercise tests; to mitigate extraneous influence on the results, the participants were also asked not to engage in moderate or intense exercise 48 h before both exercise tests.

To measure the V̇O_2_ max of the participants, we used a bicycle ergometer (839E, Monark, Varberg, Sweden) for a maximal graded exercise test. After participants sat still for 2 min, they sat on the stationary bicycle and started cycling at the speed of 70 ± 10 rpm. The participants began the exercise with a 2-min warm-up at 25 W loading, where the loading was increased by 15 W every 2 min. The testing was terminated when the participants could no longer continue the exercise due to bradypnea or fatigue, although the bicycle speed was maintained at 70 rpm. Subsequently, the participants rested for 3 min at a loading of 0 W (no resistance). Throughout the exercise testing, the participants wore a watch to monitor their heart rate and a mask to monitor their breathing. Breath-by-breath analysis was conducted on the participant data through a cardiopulmonary testing system (MetaMax 3B, Cortex, Germany). V̇O_2_ max was defined as the maximum average oxygen uptake for 20 consecutive seconds. To ensure that every participant reached V̇O_2_ max, we defined V̇O_2_ max as being reached if two of the three following conditions were met: (1) V̇O_2_ plateaus with increases in work rate; (2) the maximum respiratory exchange ratio is ≥1.10; and (3) 90% of the expected maximal heart rate, obtained by subtracting the participant’s age from 220, is reached (American College of Sports Medicine, 2009). Nearly all participants satisfied the criteria for an acceptable V̇O_2_ max, with only one participant excluded from the V̇O_2_ max test due to suspected symptoms of arrhythmia observed in the step test.

### 3MPKS test

**Figure 1:**
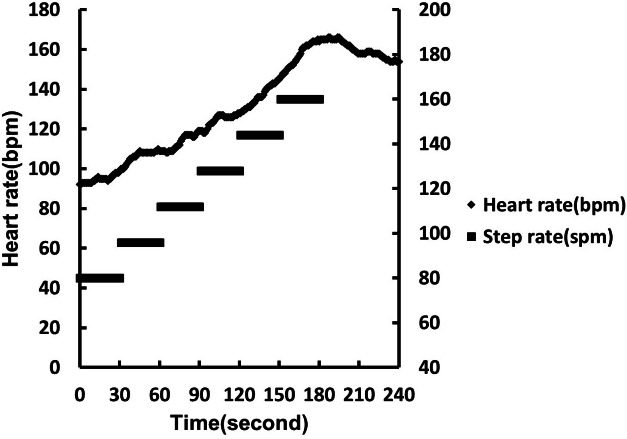
3MPKS heart rate model and corresponding step frequency.

Prior to the 3MPKS test, the participants wore a sports watch with heart rate (Polar V800, USA) and stride sensors (Polar S3 BlueTooth Stride Sensor, USA). The heart rate sensor was placed at the center of each participant’s chest using a heart rate belt (Polar H10), and the step sensor was fixed on a pair of shoes, with shoelaces, to monitor their heartbeat and number of steps taken. After the devices were worn, we measured the midpoint of the line connecting the anterior epicondyle to the midpoint of the sacrum. We marked the midpoint on the wall using colored tape as a reference for the height at which the knee should be lifted to when stepping. After the test started, the participants followed the appropriate rhythm and were required to lift their knee to the marked height at each step. The participants began the test at a pace of 80 spm (steps per minute), which increased by 16 spm every 30 s in six stages. The participants walked in stages 1 to 4 and had to perform stationary running in stages 5 and 6 (Fig. 1). We stopped the exercise if the participants could not achieve the requisite knee height or rhythm for 30 s. For their safety, the participants were asked to relax at a step rate of 80 spm in the first 30 s before resting in a standing position. We recorded the participants’ heart rate during the exercise, at the end of the exercise, and 1 min after the end of the exercise. Thirty-four participants were excluded because (1) their heart rate data were missing, (2) their heart rate was 0, (3) they did not maintain the requisite step frequency or knee height for 20 consecutive seconds, (4) they failed to complete the step test within the requisite duration, and (5) they were suspected of having heart arrhythmia. Potential predictor variables for the results of the 3MPKS test were based on per-second heart rate data collected during the test. The data included heart rate at the beginning as well as at the first, second, third, and fourth minutes, denoted by HR0, HR1, HR2, HR3, and HR4, respectively, and were used for subsequent analysis.

### Statistical analyses

To construct and subsequently evaluate a model for estimating relative oxygen uptake, we divided the full sample set (*n* = 166) into a 75% training sample set (*n* = 124) and 25% test sample set through simple random sampling. We analyzed the descriptive statistics for the main parameters, for the whole sample, and for the two subsamples.

### Development of prediction model

Using Pearson correlation coefficients, we examined the relationship between the predicted and actual relative oxygen uptakes. Multiple regression analysis was used to construct a method for selecting which variables to include in the model for predicting relative oxygen uptake. Through a backward-selection regression approach, the initial model included all possible predictors, including sex (men = 1, women = 0), age, BMI, BF%, HR0, HR1, HR2, HR3, HR4, △HR0 − HR1, △HR1 − HR2, △HR2 − HR3, △HR3 − HR0, and △HR3 − HR4. Additionally, we constructed a BMI model and BF% model to predict body composition. The goodness of fit and precision of the regression equations were evaluated using the multiple coefficient of determination (*R*^2^), absolute standard error of estimate (SEE), and relative SEE (%SEE).

To construct an accurate regression model, the regression assumptions were verified. We conducted a Kolmogorov–Smirnov test to examine the normality of the residuals, and we calculated the variation inflation factor (VIF) to check for multicollinearity.

All statistical analyses were performed using SPSS version 20 (IBM, USA). Statistical significance was indicated by an alpha level of 0.05.

## Results

The 166 participants had an average age of 41.9 ±9.6 years (range: 22–64 years), and 40% of them were men. Their mean relative oxygen uptake was 34.45 ±8.69 mL**/**kg/min. The training sample and test sample did not differ significantly with respect to their parameter values (*p* > 0.05) Table 1.

The test–retest reliability of the 3MPKS test, as evaluated in our laboratory, was excellent: the intraclass correlation coefficient (ICC) was 0.88 (95% confidence interval [CI]: 0.77–0.94), and 60 Taiwanese adults tested 1 week apart participated in this evaluation. In general, good, moderate, and poor reliability levels are indicated by ICC values of >0.75, 0.5–0.75, and <0.5, respectively.

According to the correlation matrix, V̇O_2_ max had the strongest correlation with BF% among all variables (*R* = −0.662; training data set, *n* = 124). In addition, V̇O_2_ max was significantly correlated with the heart rate parameters (HR0, HR2, HR3, and HR4), whose data were collected in the step test. V̇O_2_ max was most and least correlated with HR4 (*R* = −0.442) and HR3 (*R* = −0.289), respectively. Despite the high correlation between V̇O_2_ max and the heart rate parameters at different stages, the heart rates of the participants were expected to increase continuously from the first to third minutes of stepping, if performed properly. An individual’s heart rate typically reaches its peak immediately after exercise, and it either decreases at 1 min after exercise or does not decrease at all depending on whether the individual recovers quickly or poorly. Because heart rate is dynamic, to establish a regression model, we used combinations of heart rate parameters and adopted the difference between predicted and measured heart rate data at each stage as inputs (Table 2).

**Table 2:** Correlation between V̇O_2_max and features in training dataset (*n* = 124).

	V̇O_2_max	Sex	Age	BMI	BF%	HR0	HR1	HR2	HR3
Sex	0.597^**^								
Age	−0.342^**^	−0.114							
BMI	−0.083	0.334^**^	−0.160						
BF%	−0.662^**^	−0.491^**^	0.109	0.448					
HR0	−0.317^**^	−0.242^*^	−0.101	−0.058	0.227^*^				
HR1	−0.344^**^	−0.033	−0.283^*^	−0.039	0.274^*^	0.69^**^			
HR2	−0.357^**^	−0.312^**^	−0.093	−0.005	0.308^**^	0.592^**^	0.899^**^		
HR3	−0.289^*^	−0.254^*^	−0.21^*^	0	0.248^*^	0.525^**^	0.725^**^	0.8^**^	
HR4	−0.442^**^	−0.42^**^	−0.13	−0.063	0.334^**^	0.564^**^	0.57^**^	0.629^**^	0.702^**^

**Notes.**

BF% body fat percentage

** Correlation coefficient is significant(*p* < 0.001).

* Correlation coefficient is significant(*p* < 0.05).

The results of our other cross-validation analyses are presented in terms of CE (Constant error) values. The absolute CE values for subgroups stratified by sex and age were <1.00 for the two models (both in training and testing data sets, *n* = 124 and 42). Regarding the subgroups stratified by V̇O_2_ max, the CE values were negative in low-fitness, middle-fitness subgroups in training data set and low-fitness in testing data set. On the other hand, the CE values were positive in high-fitness in all two data sets (Table 3).

**Table 3:** Measured versus predicted V̇O_2_max constant error (CE) and standard deviations (SD) for subgroups of the training dataset and testing dataset.

Subgroup	*n*(%)	BF% model(%)			BMI model(kg m^−2^)	
		CE	SD		CE	SD
Training set(*n* = 124)						
Sex						
Female	80(64.5)	−0.01	3.95		0.01	4.45
Male	44(35.5)	−0.02	6.23		0.01	5.99
Age						
<40 years	48(38.7)	−0.34	4.72		−0.49	4.94
40–50 years	44(35.5)	0.21	5.35		0.22	5.24
≥50 years	32(25.8)	0.17	4.46		0.47	4.95
V̇O_2_max						
<29 ml/kg/min	34(27.4)	−2.77	3.19		−3.13	3.66
29–38 ml/kg/min	56(45.2)	−0.22	4.46		−0.22	4.60
≥38 ml/kg/min	34(27.4)	3.09	5.18		3.51	4.75
Testing set(*n* = 42)						
Sex						
Female	21(50)	−0.15	5.85		−0.89	5.84
Male	21(50)	0.87	6.82		0.08	7.62
Age						
<43 years	24(57.1)	0.67	6.22		−0.07	6.8
≥43 years	18(42.9)	−0.05	6.55		−0.85	6.79
V̇O_2_max						
<35 ml/kg/min	22(52.4)	−2.88	4.95		−3.86	5.31
≥35 ml/kg/min	20(47.6)	3.93	5.73		3.39	6.11

**Figure 2:**
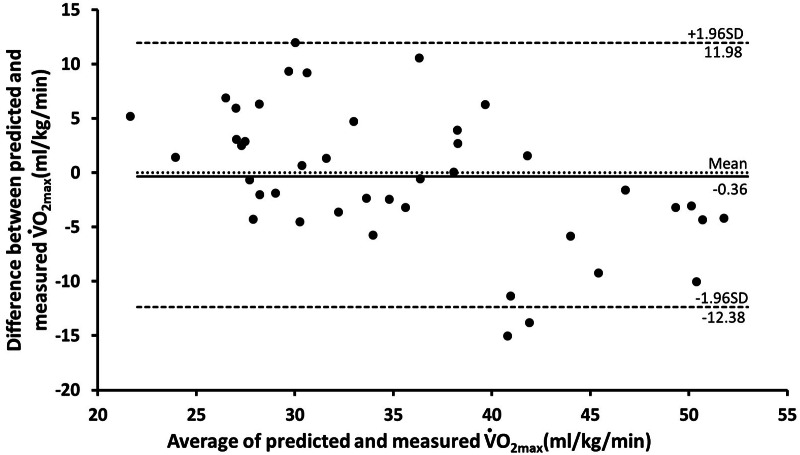
Bland Altman plot, including limits of agreement, for predicted and measured V̇O_2_ max (ml/kg/min) of BF% model by testing dataset (*n* = 42). Black line mean difference. Dashed line±1.96 ×SD.

**Figure 3:**
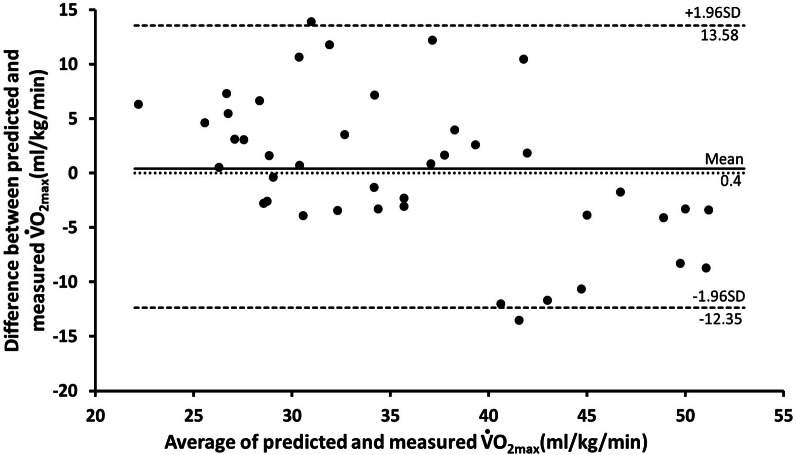
Bland Altman plot, including limits of agreement, for predicted and measured V̇O_2_ max (ml/kg/min) of BMI model by testing dataset (*n* = 42). Black line mean difference. Dashed line±1.96 ×SD.

Figures 2 and 3 present the Bland–Altman plots produced by the BF% and BMI models based on the testing data set (*n* = 42). As evident in the plots, the differences between the predicted and measured data were within an acceptable range. The mean error of the BF% model was −0.36 mL/kg/min (95% CI [−12.38–11.98]). For the BMI model, the mean error was 0.4 mL/kg/min (95% CI [−12.35–13.58]). In the BF% and BMI models, the errors for three and two participants, respectively, fell outside the 95% CI.

**Figure 4:**
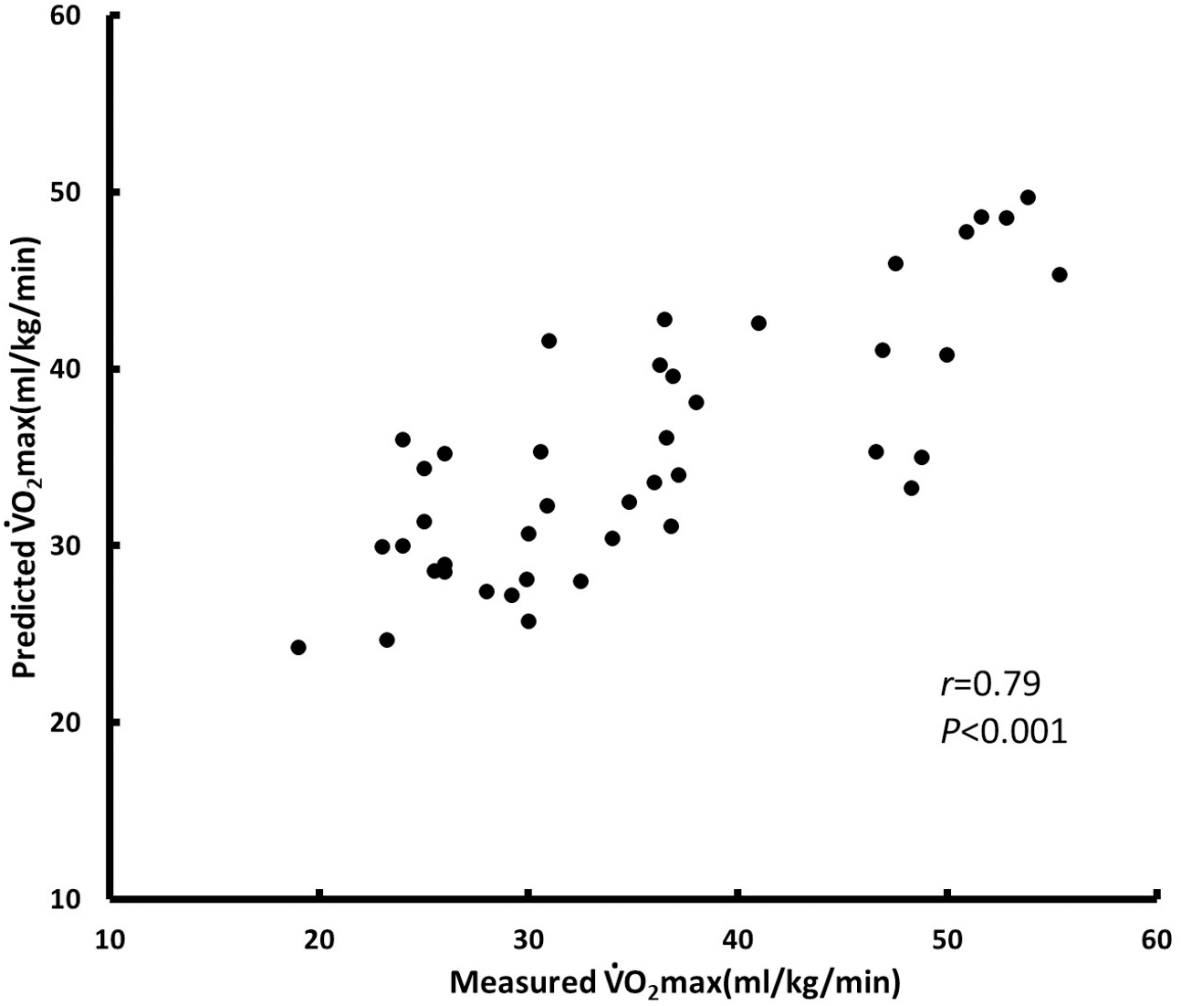
BF% model for testing test (*n* = 42).

**Figure 5:**
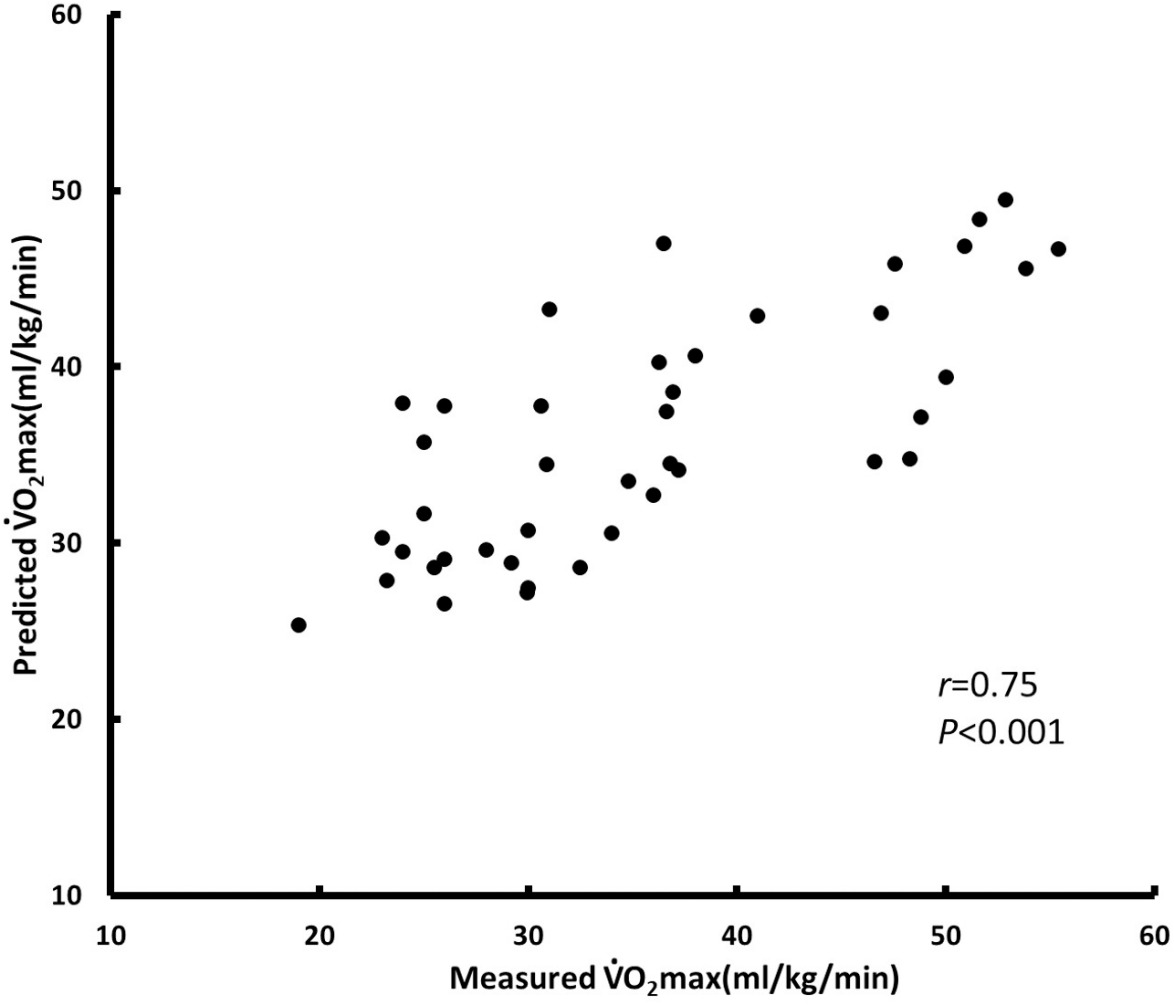
BMI model for testing (*n* = 42).

We constructed a model to predict relative oxygen uptake by using multiple regression analysis. The parameters selected for the BF% model were sex, age, BF%, HR0, ΔHR3 − HR0, and ΔHR3 − HR4; *R*^2^ = 0.624 and SEE = 4.982 (training data set, *n* = 124) (Fig. 4). The parameters selected for the BMI model were sex, age, BMI, initial heart rate, ΔHR3 − HR0, and ΔHR3 − HR4; *R*^2^ = 0.567 and SEE = 5.153 (training data set, *n* = 124) (Fig. 5). We used BF% as a predictor of body composition; it is more accurate relative to BMI, which is calculated using only height and weight (Table 4). Table 4 presents the cross-validation results for the predicted residual sum of squares (PRESS) statistics (*R*^2^*p* = 0.64 and SEE *p* = 4.84), which demonstrated minimal shrinkage in the accuracy of the regression model.

All regression assumptions were satisfied in our V̇O_2_ max prediction models. Specifically, the Kolmogorov–Smirnov test indicated normality in the residuals (*p* > 0.05). No pattern was determined in the scatter plot between the residuals and predicted V̇O_2_ max. Multicollinearity was absent among the predictor variables: the VIF ranges for the BF% and BMI models were 1.09–1.49 and 1.10–1.40, respectively; multicollinearity is absent if VIF ≤ 10 (O’brien, 2007).

## Discussion

This study developed a practical and easy-to-use model for predicting V̇O_2_ max in Taiwanese people. We recruited 166 Taiwanese adults and constructed and then evaluated a prediction model. Our results suggest that age, sex, and BF% as well as heart rate during the step test are excellent predictors of V̇O_2_ max. We also developed a novel 3MPKS test.

**Table 4:** Estimation of V̇O_2_ max through multiple regression model (*n* = 124).

	BF% model (%)				BMI model (kg m^−2^)					
V̇O_2_max (ml kg^−1^ min ^−1^)	Coefficients	*β*	*p* value		Coefficients	*β*	*p* value			
Constant	72.334		.000		82.387		.000			
Sex (0=women, 1=men)	4.366	0.258	.000		9.338	0.551	.000			
Age(yr)	−0.261	−0.302	.000		−0.327	−0.378	.000			
Body composition	−0.448	−0.421	.000		−0.718	−0.346	.000			
HR0	−0.134	−0.214	.001		−0.171	−0.273	.001			
ΔHR3- HR0	−0.082	−0.136	.041		−0.099	−0.163	.017			
ΔHR3-HR4	0.073	0.124	.048		0.081	0.139	.032			
R^2^	0.624				0.567		
SEE	4.982				5.153		
SEE%	14.46				14.96		
PRESS	2904.186				3107.325		
SEEp	4.840				5.006		
R^2^p	0.644				0.619		

**Notes.**

BMI body mass index BF% body fat percentage *β* standardized regression weights SEE standard error of estimate SEE% SEE / mean of measured V̇O_2_ max ×100. PRESS predicted residual error sum of squares SEEp PRESS standard error of estimate *R*^2^*p* PRESS squared multiple correlation coefficient

Nes et al. (2011) conducted large-scale V̇O_2_ max tests on 4,260 participants. They developed a nonexercise model and determined four variables (age, waist circumference, physical activity, and resting heart rate) to be excellent predictors of V̇O_2_ max; for their model, *R*^2^ was 0.61 and SEE was 5.70 mL/kg/min for men, and *R*^2^ was 0.56 and SEE was 5.14 mL/kg/min for women. Jackson et al. (2012) conducted a 27-year study that examined the V̇O_2_ max of 11,365 people and used variables such as age, sex, BMI, waist circumference, resting heart rate, physical activity, and smoking habits to estimate CRF; for their model, *R* was 0.78–0.81 and SEE was 5.3–5.6 mL/kg/min. Although the nonexercise model is an excellent predictor of V̇O_2_ max, its SEE is generally higher than those of submaximal exercise models; compared with nonexercise models, our developed BF% model had better predictive performance and a lower standard error of estimate (*R*^2^ = 0.624 and SEE = 4.982). Abut, Akay & George (2016) reported that (1) when perceived functional ability (PFA) was used as the sole predictor of V̇O_2_ max, an *R* value of 0.73 and a higher RMSE of 6.08 mL/kg/min could be obtained; (2) when submaximal ending speed (SM-ES) of a treadmill was used as the sole predictor, the *R* value increased to 0.82 and the RMSE was relatively low at 4.99 mL**/**kg/min; and (3) when both PFA and SM-ES were used as predictors, the *R* value was 0.89 and RMSE was 4.14 mL**/**kg/min. These findings indicate that predicted values of V̇O_2_ max that are based only on participant self-reports are likely to deviate from their measured values. Although predictive performance is ostensibly improved when motion is added to the prediction model, the cost of exercise tests due to the use of this method restricts its application in large-scale tests.

Several studies have developed simple models involving submaximal motion. Lee et al. (2019) investigated 568 adults and used sex, age, height, and weight and inverse recovery heart rate during a YMCA step test to predict V̇O_2_ max; for their model, *R* was 0.78 and SEE was 4.74 mL/kg/min. The duration of their exercise test plus recovery time was only 4 min, and they used exercise-induced heart rate as a predictor; their results are similar to ours. Their study provided a simple and practical method for simultaneously estimating CRF in many Korean adults. Cao et al. (2013) used age, sex, and physical composition as well as stepping distance over a 3-min period to develop a set of prediction methods. They determined that BF% (a measure of body composition) was a better predictor than BMI (*R*^2^ = 0.83 vs. 0.80, SEE = 4.565 vs. 5.037 mL/kg/min). In contrast to our method, their method has the considerable advantages of a shorter testing time of 3 min and the fact that participants need not wear a heart rate monitor. However, their test is limited by its need for a 20-m open space. Similarly, we found that sex, age, and BF% as well as heart rate during the 3MPKS test yielded the best prediction performance (*R* = 0.79, SEE = 4.982 mL/kg/min). Because BMI is based on only height and weight and may not accurately represent the body characteristics of participants, BMI is a less accurate predictor than BF%.

Most submaximal exercise models proposed by previous studies involve a fixed-height step test. However, the height and leg length of participants when standing may affect their physiological response in the step test (Culpepper & Francis, 1987). Relative to their European counterparts, Asian adults have shorter heights and leg lengths when standing (Stanfield et al., 2012). Therefore, differences in heart rate and oxygen consumption potentially affect the model’s prediction. The 3MPKS test employs the knee-ups and step test to measure the physical fitness and cardiopulmonary endurance of older adults (Rikli & Jones, 2001). In the test, participants must execute tasks at various knee heights based on their thigh length, and individualized exercise testing goals are provided. Moreover, most field tests involve average speed tests, such as step tests and running. In running tests specifically, if the distance is used as the capacity index but the speed or frequency of exercise is not progressively increased, participants may exercise intensely at the beginning of the test (i.e., run at a higher speed). However, due to the lack of appropriate speed allocation, decremental loading occurs in participants as their physical strength decreases. The difficulty of diagnosing potential heart diseases in advance increases the risk of sudden death during running tests. To the best of our knowledge, research has not been conducted on the ethics of running tests. Most previous studies have investigated the rate of sudden death among athletes in long-distance competitions. However, cases of sudden cardiac death occur frequently worldwide during running tests, and the principle that physical activities ought to be progressive must be adhered to in physical fitness tests. Our research method used body composition and heart rate as variables. The advantages of the 3MPKS test are that it does not require a step-up box and is not subject to venue restrictions. These make the 3MPKS test accord with the principle that physical activities ought to be progressive, thus making it safer.

Considering the immediacy of heart rate measurement and that of confounding factors, we used a chest-worn heart rate monitor in the experiment. Although the requirement of heart rate monitoring constitutes a disadvantage for the 3MPKS test, it is ameliorated by the prevalence of low-cost wearable devices. More comfortable than the chest-worn heart rate belt, products that combine running clothes with heart rate belts have also appeared on the market. Research has also suggested a high correlation between the heart rate measurements of various types of optical devices and chest-worn heart rate belts (Stahl et al., 2016). Therefore, when conducting a large-scale cardiorespiratory general test, the use of easily wearable optical heart rate monitors can be considered. The whole-range monitoring of heart rate can also considerably improve test safety in a field study. Notably, through whole-range monitoring, we found that one research participant was likely to have an unknown heart disease. We then terminated the experiment for the participant and recommended that the participant seek medical treatment. This example illustrates a side benefit of CRF tests.

In our research model, heart rate during stepping at each stage was used as the main variable. Therefore, the test may be unsuitable for individuals who have psychological sensitivity or dysautonomia or who are taking medication. Furthermore, because our participants were adults between 20 and 64 years old, it was unclear whether our 3MKPS test is appropriate as a physical fitness and cardiorespiratory test for students (7–23 years old) and older adults (≥65 years old). Future research must include samples with greater diversity in age and ethnicity to assess whether our 3MKPS test can be applied to the wider global population.

## Conclusion

This study, involving Taiwanese adults, constructed and verified a model for predicting V̇O_2_ max, which is used to measure CRF. This model comprises the predictors sex, age, and body composition as well as heart rate changes during a step test. Our 3MKPS test has three advantages: it has a short testing time of 4 min, it has no venue limitations, and it does not require a step box. Furthermore, measurements can be taken for many participants simultaneously by asking them to wear a heart rate monitor and move according to a beat. Our model can also be applied to large-scale epidemiological research. In future applications, the model can be combined with smartwatches or used to develop health and well-being apps, helping users to track their V̇O_2_ max. Future research can further explore the correlation between various diseases and V̇O_2_ max, as predicted using our simple and reliable method for measuring CRF.

##  Supplemental Information

10.7717/peerj.10831/supp-1 Supplemental Information 1 Raw data

10.7717/peerj.10831/supp-2 Supplemental Information 2 Codebook

## Additional Information and Declarations

### Data Availability

The following information was supplied regarding data availability: Raw measurements are available in the Supplemental Files.

## References

[ref-1] Abut F, Akay MF, George J (2016). Developing new VO_2_ max prediction models from maximal, submaximal and questionnaire variables using support vector machines combined with feature selection. Computers in Biology and Medicine.

[ref-2] American College of Sports Medicine (2009). ACSM’s guidelines for exercise testing and prescription.

[ref-3] Beutner F, Ubrich R, Zachariae S, Engel C, Sandri M, Teren A, Gielen S (2015). Validation of a brief step-test protocol for estimation of peak oxygen uptake. European Journal of Preventive Cardiology.

[ref-4] Blair SN, Kohl HW, Paffenbarger RS, Clark DG, Cooper KH, Gibbons LW (1989). Physical fitness and all-cause mortality: a prospective study of healthy men and women. Journal of the American Medical Association.

[ref-5] Buckley JP, Sim J, Eston RG, Hession R, Fox R (2004). Reliability and validity of measures taken during the Chester step test to predict aerobic power and to prescribe aerobic exercise. British Journal of Sports Medicine.

[ref-6] Cao ZB, Miyatake N, Aoyama T, Higuchi M, Tabata I (2013). Prediction of maximal oxygen uptake from a 3-minute walk based on gender, age, and body composition. Journal of Physical Activity and Health.

[ref-7] Chang SC, Lin JC (1995). The validity generalization of step test as a measure of the maximal oxygen intake. Physical Education Journal.

[ref-8] Culpepper MI, Francis KT (1987). An anatomical model to determine step height in step testing for estimating aerobic capacity. Journal of Theoretical Biology.

[ref-9] Grant JA, Joseph AN, Campagna PD (1999). The prediction of VO_2_max: a comparison of 7 indirect tests of aerobic power. Journal of Strength and Conditioning Research.

[ref-10] Jackson AS, Sui X, O’Connor DP, Church TS, Lee DC, Artero EG, Blair SN (2012). Longitudinal cardiorespiratory fitness algorithms for clinical settings. American Journal of Preventive Medicine.

[ref-11] Jiménez-Pavón D, Lavie CJ, Blair SN (2019). The role of cardiorespiratory fitness on the risk of sudden cardiac death at the population level: a systematic review and meta-analysis of the available evidence. Progress in Cardiovascular Diseases.

[ref-12] Kaminsky LA, Arena R, Beckie TM, Brubaker PH, Church TS, Forman DE, Franklin BA, Gulati M, Lavie CJ, Myers J, Patel MJ, Piña IL, Weintraub WS, Williams MA (2013). The importance of cardiorespiratory fitness in the United States: the need for a national registry: a policy statement from the American Heart Association. Circulation.

[ref-13] Kodama S, Saito K, Tanaka S, Maki M, Yachi Y, Asumi M, Sugawara A, Totsuka K, Shimano H, Ohashi Y, Yamada N, Sone H (2009). Cardiorespiratory fitness as a quantitative predictor of all-cause mortality and cardiovascular events in healthy men and women: a meta-analysis. Journal of the American Medical Association.

[ref-14] Laukkanen JA, Kurl S, Salonen R, Rauramaa R, Salonen JT (2004). The predictive value of cardiorespiratory fitness for cardiovascular events in men with various risk profiles: a prospective population-based cohort study. European Heart Journal.

[ref-15] Lee O, Lee S, Kang M, Mun J, Chung J (2019). Prediction of maximal oxygen consumption using the Young Men’s Christian Association-step test in Korean adults. European Journal of Applied Physiology and Occupational Physiology.

[ref-16] Mazic S, Zivotic-Vanovic M, Igracki I, Zivanic S, Velkovski S (2001). A simple and reliable step-test for indirect evaluation of aerobic capacity. Medicinski Pregled.

[ref-17] McLester CN, Nickerson BS, Kliszczewicz BM, McLester JR (2020). Reliability and agreement of various InBody body composition analyzers as compared to dual-energy X-ray absorptiometry in healthy men and women. Journal of Clinical Densitometry.

[ref-18] Nes BM, Janszky I, Vatten LJ, Nilsen TIL, Aspenes ST, Wisløff U (2011). Estimating VO 2peak from a nonexercise prediction model: the HUNT study, Norway. Medicine & Science in Sports & Exercise.

[ref-19] O’brien RM (2007). A caution regarding rules of thumb for variance inflation factors. Quality and Quantity.

[ref-20] Rikli RE, Jones CJ (2001). Senior fitness test manual.

[ref-21] Ross R, Blair SN, Arena R, Church TS, Després JP, Franklin BA, Haskell WL, Kaminsky LA, Levine BD, Lavie CJ, Myers J, Niebauer J, Sallis R, Sawada SS, Sui X, Wisløff U (2016). Importance of assessing cardiorespiratory fitness in clinical practice: a case for fitness as a clinical vital sign: a scientific statement from the American Heart Association. Circulation.

[ref-22] Sawada SS, Lee IM, Naito H, Kakigi R, Goto S, Kanazawa M, Okamoto T, Tsukamoto K, Muto T, Tanaka H (2014). Cardiorespiratory fitness, body mass index, and cancer mortality: a cohort study of Japanese men. BMC Public Health.

[ref-23] Stahl SE, An HS, Dinkel DM, Noble JM, Lee JM (2016). How accurate are the wrist-based heart rate monitors during walking and running activities? Are they accurate enough?. BMJ Open Sport & Exercise Medicine.

[ref-24] Stanfield KM, Wells JC, Fewtrell MS, Frost C (2012). Differences in body composition between infants of South Asian and European ancestry: the London Mother and Baby study. International Journal of Epidemiology.

[ref-25] Su MY, Lin CC, Hsieh SY (2006). The validity and reliability of a step test with adjusted heights according to leg lengths. Sports & Exercise Research.

[ref-26] Sui X, LaMonte MJ, Blair SN (2007). Cardiorespiratory fitness as a predictor of nonfatal cardiovascular events in asymptomatic women and men. American Journal of Epidemiology.

[ref-27] Yoopat P, Vanwonterghem K, Louhevaara V (2002). Evaluation of a step-test for assessing the cardiorespiratory capacity of workers in Thailand: a pilot study. Journal of Human Ergology.

