# Supplementary material for: Predicting maximal oxygen uptake from a 3-minute progressive knee-ups and step test
Source: PeerJ. 2021 Mar 15;9:e10831. doi: 10.7717/peerj.10831 (PMC7971079; doi:10.7717/peerj.10831)
Supplement: Supplemental Information 2 [file peerj-09-10831-s002.docx]

**codebook**

| **Variable** | **Description** |
| --- | --- |
| data set | training set or testing set |
| no. | serial number for participants |
| age | the age of participants(year) |
| gender | the gender of participants 0：women、1：men |
| height | the height of participants(cm) |
| weight | the weight of participants(kg) |
| body fat | body fat percentage (BF%) |
| HR0 | heart rate at the beginning(bpm) |
| HR2 | heart rate at the second minute(bpm) |
| HR3 | heart rate at the third minute(bpm) |
| HR4 | heart rate at the fourth minute(bpm) |
| V02 max | the value of the maximum oxygen uptake(ml/kg/min) |
